# Isolated bone marrow amyloid light chain amyloidosis coexisting with multiple myeloma

**DOI:** 10.1002/jha2.631

**Published:** 2023-03-13

**Authors:** José R. Álamo Moreno, Paola Castillo, Estella Matutes

**Affiliations:** ^1^ Department of Haematopathology Hospital Clinic de Barcelona Barcelona Spain; ^2^ Department of Pathology Hospital Clinic de Barcelona Barcelona Spain

1

A 62‐year‐old woman was diagnosed with Bence‐Jones protein multiple myeloma in another hospital. She referred sternum pain and a clavicle fracture. Nuclear magnetic resonance showed multiple lytic lesions in clavicle, rib, vertebra, humerus and sternum. She was referred to our hospital because of an inadequate response to treatment. Complete blood count, kidney function, calcium, lactate dehydrogenase and beta‐2‐microglobulin did not show abnormalities. Monoclonal immunoglobulins in serum were not detected. Immunofixation in serum and urine was positive for kappa light chain. A bone marrow aspirate was performed in the sternum.

The sternal aspirate showed a patchy infiltration by plasma cells (CD19^–^, CD56^+^, CD117^+^) of variable size, some of them binucleated with a cytoplasm with a flammig appearance. Nebulous amorphous basophilic protein material suggestive of amyloid was also observed (Figure 1a). Congo red stain was positive, and the microscope fluorescence showed apple‐green birefringence under polarized light and confirmed the suspicion (Figure 1b). Fluorescence in situ hybridisation (FISH) showed the t (11;14) (q13;q32); the *TP53* was normal. The study in the fat tissue did not show amyloid and the renal and heart functions were normal.

The patient was diagnosed of amyloid light chain amyloidosis associated with multiple myeloma and started treatment with bortezomib, lenalidomide and dexamethasone. The response was suboptimal, and daratumumab was added to the treatment.

This case demonstrates the importance of recognizing the presence of amyloid in bone marrow aspiration. This diagnosis is relevant for the prognostic and therapeutic implication.

**FIGURE 1:**
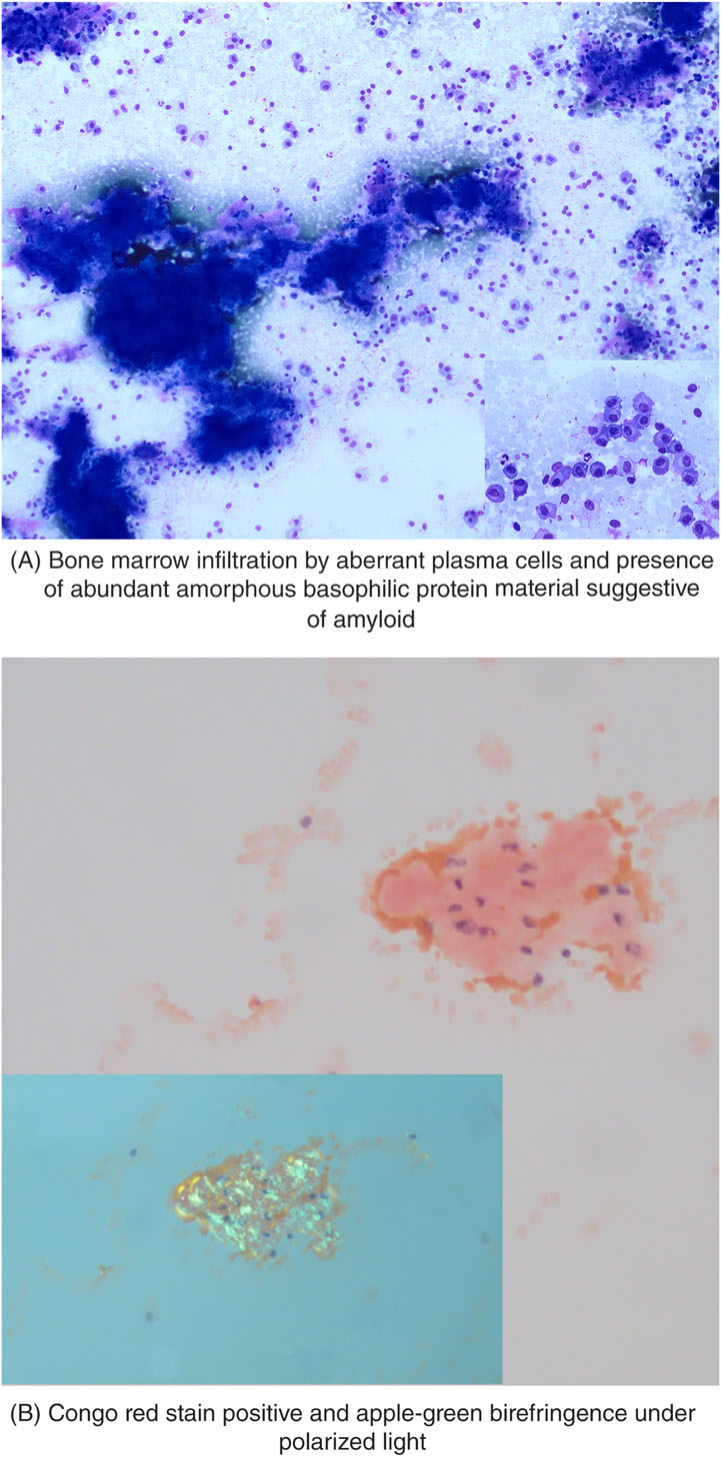
Bone marrow aspiration: May Grunwald‐Giemsa stain (1a) and Congo red stain (1b).

## AUTHOR CONTRIBUTIONS

All authors have contributed equally to the manuscript.

## CONFLICT OF INTEREST

The authors declare they have no conflicts of interest.

## FUNDING INFORMATION

The authors received no specific funding for this work.

## PATIENT CONSENT STATEMENT

A written informed consent was obtained from the patient.

